# Molecular Markers with Predictive and Prognostic Relevance in Lung Cancer

**DOI:** 10.1155/2012/729532

**Published:** 2012-09-19

**Authors:** Alphy Rose-James, Sreelekha TT

**Affiliations:** Laboratory of Biopharmaceuticals, Division of Cancer Research, Regional Cancer Centre, Kerala, Trivandrum 695011, India

## Abstract

Lung cancer accounts for the majority of cancer-related deaths worldwide of which non-small-cell lung carcinoma alone takes a toll of around 85%. Platinum-based therapy is the stronghold for lung cancer at present. The discovery of various molecular alterations that underlie lung cancer has contributed to the development of specifically targeted therapies employing specific mutation inhibitors. Targeted chemotherapy based on molecular profiling has shown great promise in lung cancer treatment. Various molecular markers with predictive and prognostic significance in lung cancer have evolved as a result of advanced research. Testing of EGFR and Kras mutations is now a common practice among community oncologists, and more recently, ALK rearrangements have been added to this group. This paper discusses various predictive and prognostic markers that are being investigated and have shown significant relevance which can be exploited for targeted treatment in lung cancer.

## 1. Introduction

Lung cancer is the leading cause of cancer-related deaths in both men and women worldwide. The upward trend in lung cancer mortality is due to lack of significant markers for early detection and treatment. Lung cancer, the leading cancer killer among men and women worldwide, is considered to be a deadly illness because of low proportion of subjects (*≈*15%) who are still alive 5 years after the initial diagnosis. This low 5-year survival rate is mainly because most of subjects present with advanced stages at the time of diagnosis. Diagnosing lung cancer at localized early stage increases the 5-year survival rate significantly. Advances in molecular biology have eased the systematic efforts to identify molecular markers for lung cancer with valuable predictive and prognostic significance. It is estimated that around 10–20 genetic events including alterations in oncogenes and tumor suppressor genes (TSG) will have been occurred by the time a lung tumor becomes clinically evident [1]. These alterations if studied and characterized systematically using the present day advanced molecular analytic techniques can be developed as potential predictive and prognostic markers. Moreover, by understanding molecular mechanisms of the disease and potential treatment, molecularly targeted treatment strategies can be adopted. Many attempts have been undertaken by scientists worldwide to identify potential biomarker for lung cancer—the world no. 1 among cancer killers.

## 2. Molecular Markers in Lung Cancer

**Figure 1:**
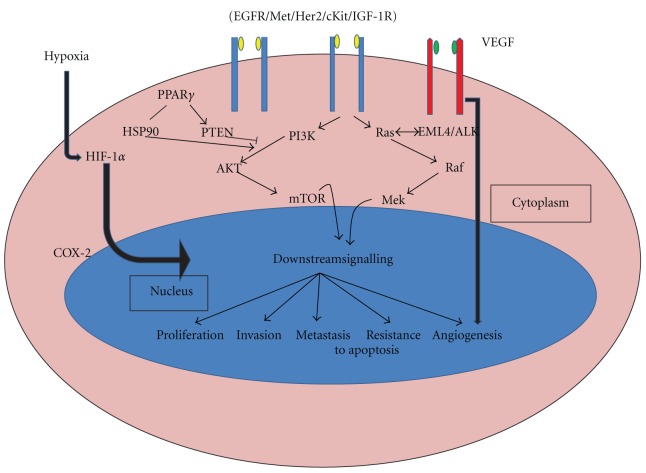
Major signalling pathways affected by altered genes in lung cancer.

A molecular marker for cancer can be defined as a molecular entity (DNA, RNA, or protein) which can be isolated from biological materials, give quantifiable measurements of biological homeostasis, and indicates cancer-specific alterations in physiology from the normal state. Tumor markers can be broadly classified into 2:* Prognostic markers*—those which indicate a better or worse outcome irrespective of treatment; *Predictive markers*—those which indicate better or worse treatment outcome. Identification of these markers is the major goal of translational research and forms the basis of personalized medicine as these markers say ahead of time which treatment will or will not work in a specific patient. Genetic instability is at prime position in carcinogenesis and is indicated by a variety of cellular features at the chromosomal and DNA levels. DNA instability may be due to point mutations (deletions or insertions), recombination, gene amplification, and microsatellite instability. Instability at chromosomal level is mainly manifested by aneuploidy, translocations, deletions, sister chromatid recombination, fragile sites, homogenously stained regions, and double minute chromosomes [2]. Molecular alterations that occur during lung carcinogenesis or any other cancer result in dysregulation of signalling pathways critical for cell growth and apoptosis. The major pathway affected by the molecular alterations is the PI3K/AKT/mTOR pathway. The major genes targeted in cancer are the proto-oncogenes and tumor suppressor genes. The various oncogenes and tumor suppressors that are altered in lung cancer and their role in cell signaling pathways (Figure 1) explain their molecular contributions in carcinogenesis.

**Figure 2:**
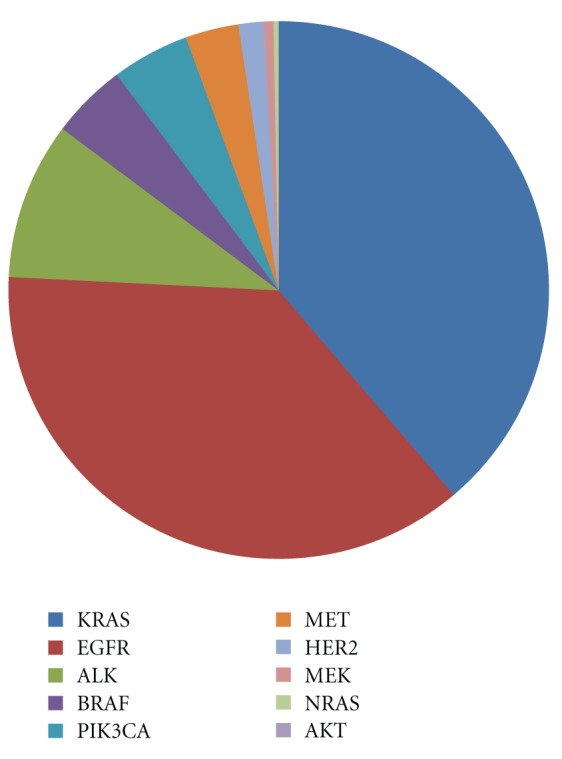
Mutation spectra in lung adenocarcinoma.

**Figure 3:**
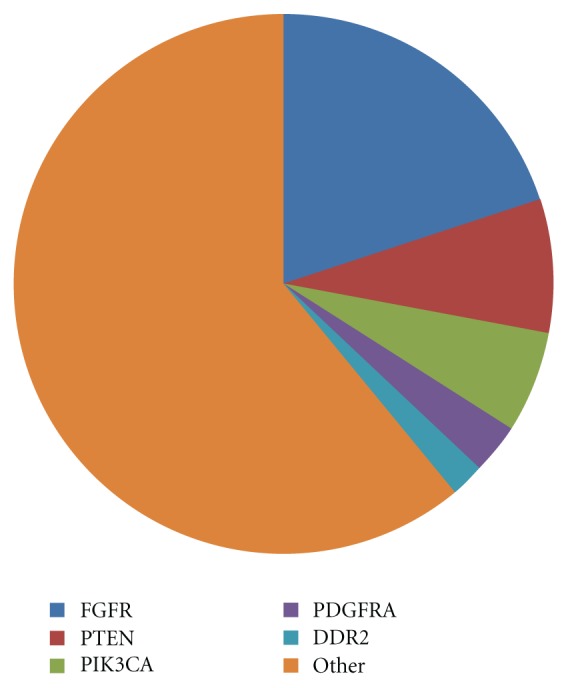
Mutation spectra in squamous cell carcinoma.

Though any one of the subtypes (SCLC or NSCLC) of lung cancer may be more susceptible to a certain type of mutation, many of the gene alterations that occur in lung cancer are common to both small-cell lung cancer (SCLC) and non-small-cell lung cancer (NSCLC). Lung cancer manifests many numeric and structural cytogenetic abnormalities. The latter include nonreciprocal translocations and recurrent losses involving 1p, 3p, 6q, 9p, 11p, 15p, and 17p, representing changes in known and potential TSGs. Aberrant DNA hypermethylation at cytosine residues within CpG islands, clustered around the promoters of many genes, is an alternative to gene mutations as a mechanism of silencing TSG expression. Polysomies or regions of gene amplifications often involve proto-oncogenes such as epidermal growth factor (EGFR) and myelocytomatosis (MYC) [3, 4]. Loss of imprinting or hypomethylation in the promoter region, of the insulin-like growth factor 2 (ILGF2), paternally expressed gene 1/mesoderm-specific transcript homolog protein (PEG1/MEST), and the H19 genes was also reported in lung cancer, suggesting that methylation is used not only to silence TSG, but also to activate potential oncogenes through hypomethylation [5, 6]. Simple reciprocal translocations are rarely observed in lung cancer, although t(15;19) has been reported [7, 8]. Alterations in microsatellites are another type of instability. The underlying mechanisms for this chromosomal instability are not yet ascertained. However, it is not surprising that the most powerful tumor surveillance mechanism is involved in DNA damage response and correction of errors in DNA replication [9]. The major driver mutations in lung adenocarcinoma and squamous cell carcinoma are represented in Figures 2 and 3 [10].

Lung carcinogenesis is a prolonged process which results in accumulation of molecular abnormalities. Genetic instability can be exploited as potential lung cancer biomarkers, but no biomarkers have adequate sensitivity, specificity, and reproducibility. Traditionally and till date, lung cancer treatment was decided based on histological subtyping into SCLC and NSCLC of which the latter is again subdivided into adenocarcinoma and squamous cell carcinoma and large cell carcinoma. With the completion of HGP and rapid advancement in molecular biology techniques, physicians have been able to delve into the molecular basis of the disease, and the concept of targeted therapy has gained popularity. Clinically relevant molecular subsets are being identified which are governed by driver mutations in genes crucial for cell proliferation and survival. For example, NSCLC can be divided into various subsets based on the driver mutations involved, like those with mutations in KRAS, EGFR, echinoderm microtubule-associated protein like 4-anaplastic lymphoma kinase (EML4-ALK), herceptin 2 (HER2), v-raf murine sarcoma (BRAF), mesenchymal epithelial transcription factor (Met), protein kinase B (PKB/AKT1), phosphatidyl inositide 3 kinase catalytic subunit (PI3KCA), and so forth, wherein EGFR mutation subset can again be divided into 3: EGFR mutations associated with drug sensitivity, EGFR mutations associated with primary drug resistance and EGFR mutations associated with acquired drug resistance [11]. This molecular subtyping would throw more light on personalized medicine which focuses on giving “right medicine for the right patient at the right time.”

Though much work has been systematically focused on lung cancer markers, the acceptance of these markers being used as part of any of the diagnostic or prognostic procedures in lung cancer patients has been limited. This paper discusses the various molecular alterations seen in lung cancer that are and can be exploited as valuable markers for diagnosis, staging, and prognosis of lung cancer.

### 2.1. EGFR

Epidermal growth factor receptor (EGFR) is the cell surface receptor for EGF family of extracellular protein ligands. The receptors exist as inactive monomers, which dimerise after ligand activation. This causes dimerisation between EGFR and other members of the Erb receptor family. After ligand binding, the Tyr kinase intracellular domain of the receptor is activated and undergoes autophosphorylation which initiates a cascade of intracellular events. EGFR signaling is not only critical for cell proliferation, but also to processes that are crucial for cancer progression, including angiogenesis, metastasis, and inhibition of apoptosis. The tyrosine kinase (TK) is the part of the protein located inside the cell, which switches on when a growth factor or ligand from outside of the cell binds to the outside protein of the EGFR. This switch when turned on allows the EGFR to signal the cells to grow and survive. In cancer patients who have mutations in the TK domain of EGFR, very little growth factor is needed to flip on the switch, and once turned on, the cancer cell is driven to grow and proliferate through this 1 signal. EGFR mutations have been widely reported in lung cancer by many researchers and play a key role in lung cancer therapy. About 10% of patients with NSCLC in the US and 35% in Asia have tumor-associated EGFR mutations [12]. These mutations occur within the EGFR exons 18–21, which encode a portion of the EGFR kinase domain [12]. EGFR mutations are usually heterozygous, with the mutant allele also showing gene amplification [13]. About 90% of these mutations are exon 19 deletions or exon 21 L858R point mutations [14] which increase the kinase activity of EGFR, leading to hyperactivation of downstream signaling pathways that promote cell survival. This is one among the mutations widely accepted for gene testing in lung cancer.

EGFR mutations are often found in tumors of female never smokers with adenocarcinoma [12]. In most of the cases, EGFR mutations are nonoverlapping with other oncogenic mutations in lung cancer. EGFR mutations are caused by carcinogens other than those found in tobacco smoke. It is recognized that EGFR-TK domain mutations represent the first molecular change occurring specifically in never smokers [12].

The understanding of EGFR status in lung cancer and the development of gefitinib is a milestone in personalized therapy in lung cancer. From the studies of Giovannetti et al. [15], it was determined that EGFR activating mutations significantly correlated with response time, longer periods of progression and overall survival in EGFR tyrosine kinase inhibitor (TKI) therapy. While patients with mutations are most likely to have a dramatic response to EGFR TKI therapy, EGFR amplification and protein expression have also been found to correlate with survival after EGFR TKI therapy. Some studies indicated that EGFR amplification and/or protein expression are better predictors of survival after EGFR TKI therapy than are mutations.

EGFR mutation and high gene copy number predicted sensitivity to EGFR TKI in advanced disease, whereas increased expression of EGFR conferred higher response to EGFR TKIs. While EGFR mutations predicted better prognosis in untreated patients, high gene copy number was associated with worse prognosis [16].

### 2.2. RAS

RAS genes (KRAS, NRAS, and HRAS) encode a family of membrane bound 21 KD GTP binding proteins that regulate cell growth, differentiation, and apoptosis by interacting with multiple effectors including those in the MAPK, STAT, and PI3K signaling cascades. Ras proteins acquire oncogenic potential when amino acid at positions 12, 13, or 61 is replaced as a result of a point mutation in the gene. This point mutation leads to constitutive activation of Ras signaling pathway. RAS mutations are prevalent in all human malignancies, but of which KRAS is most common. KRAS accounts for 90% of RAS mutations in lung adenocarcinoma, and 97% of KRAS mutations in NSCLC involve codon 12 or 13 [17]. KRAS mutations are uncommon in squamous cell carcinoma of the lung [18]. 

KRAS mutations are strongly associated with tobacco smoke. Incidence of KRAS mutations increased along with increasing smoking exposure, in contrast to the tendency for EGFR mutations [19]. While some mutations in KRAS are associated with cigarette smoking, KRAS mutations do occur in never smokers. KRAS mutations constitute a prognostic marker for poor overall survival in NSCLC patients [19]. It has been indicated as negative predictor of benefit from erlotinib or gefitinib treatment both of which are employed in anti-EGFR therapy and also in adjuvant chemotherapy [20]. 

Studies by Mills et al. [21] have proved that sensitive detection of KRAS codon 12 mutations in bronchioalveolar lavage can serve as an important aid to cytology in the diagnosis of lung cancer. Detection of these mutations could lead to earlier cancer diagnosis and less need for invasive diagnostic procedures. The role of KRAS as either a prognostic or predictive factor in NSCLC is uncertain at this time. Very few prospective randomized trials have been completed using KRAS as a biomarker to stratify therapeutic options in the metastatic setting. Unlike in colon cancer, KRAS mutations have not yet been proved in NSCLC to be negative predictors of benefit to anti-EGFR antibodies. However, KRAS mutations are negative predictors of radiographic response to the EGFR tyrosine kinase inhibitors, erlotinib and gefitinib [20]. KRAS mutations conferred resistance to treatment with EGFR TKI in advanced diseases and were found to be associated with lack of benefit from adjuvant chemotherapy in early disease [16]. However, studies focused on the prognostic significance of RAS genes were highly contradictory. KRAS mutations were found to be negative prognostic marker for adenocarcinomas, but the same did not hold true for squamous cell carcinoma [22]. Camps et al. [23] showed no prognostic value for KRAS, whereas Rosell et al. [24] had found KRAS to be a negative prognostic marker for disease relapse and morbidity for all stages and histological types. KRAS is downstream of EGFR, and hence tumors that contain KRAS mutations were found to be resistant to EGFR TKIs which makes KRAS a good marker for patients who should be excluded from EGFR TKI treatment [25] and are accepted for gene testing of lung cancer.

Somatic mutations in NRAS have been found in *≈*1% of NSCLC and are common in squamous cell carcinoma [18]. Most of the NRAS mutations are missense mutations by amino acid substitution at position 61. NRAS mutations are usually nonoverlapping with other oncogenic mutations [18]. Specific clinical characteristics of lung cancer patients harboring NRAS mutations have yet to be described. Currently, there are no direct anti-NRAS therapies available.

### 2.3. P53

P53 is a DNA binding sequence-specific transcription factor that activates the p21 TS gene which hinders the transition from G1 to S phase of the cell cycle by creating protein complexes of p21 oncoprotein with the D1/CdK4 and E/CdK2 cyclin/CdK complexes. P21 activation leads to the deactivation of the CdKs and therefore to the activation of the Rb TS gene. P53 mutation in lung cancer is widely reported. Loss of function of P53 TS gene, because of missense mutations that cause single-residue change in the DNA binding core domain of the protein, occurs early in lung tumorigenesis in about 50% of cases [26]. Genetic instability due to the impaired ability of the p53-regulated DNA damage repair further facilitates the occurrence new genetic abnormalities, leading to malignant progression [2]. Not only P53 mutations result in the abrogation of wild-type p53 activity, but the expressed p53 mutant proteins also tend to gain oncogenic functions such as interference with wild-type p53-independent apoptosis [27].

One major genetic alteration detected in lung cancer includes point mutations in the P53 gene. The rate of P53 mutations in NSCLC varies from 30% to 50% of the cases and is clustered at codons 157, 158, 248, 249, 273, and 282 within exons 5 and 8 [28–30]. These types of mutations are typical for both smokers and nonsmokers. Missense mutations of the P53 gene are most common and usually but not always prolong the half-life of the proteins from minutes to hours. This results in nuclear accumulation of the p53 protein which can be detected by immunohistochemistry (IHC) [31]. It has been shown that p53 mutations in lung cancer are different from those in other cancers and that an excess of G to T transversions is characteristic of these tumors [32, 33]. The genetic profile of p53 mutations in lung cancer and particularly in adenocarcinoma differs according to their characteristics and location.

Cherneva et al. [34] have reported high incidence of p53 overexpression in tumor tissues of patients with NSCLC. This was associated with the overexpression of p53 mRNA in corresponding lymph nodes and a trend to be associated with the advanced stages. The expression analysis of p53 mRNA, detected by real-time PCR, can supplement the knowledge of p53 as a biomarker of lung cancer diagnosis and pathogenesis. This method is more sensitive than the currently used methods for p53 expression analysis and thus provides opportunities for a more accurate clinical application of molecular markers.

In lung cancer, the prevalence of p53 Ab is high (30%) and is correlated with a very high rate of P53 mutations in this cancer (60–70%). Lubin et al. [35] showed that these Ab are always present at the time of diagnosis, but they never appear during tumor development, an observation strengthened by the fact that these Ab are mostly IgG, corresponding to secondary immune response. These results suggest that the humoral response is an early event and that p53 Ab can be used as a precocious marker of p53 alterations before clinical manifestation of the disease.

According to the study by C. J. Piyathilake, p53 accumulation is an early event in lung carcinogenesis and potentially could be useful in the identification of smokers who are at risk of developing SCC, but not in the estimation of survival of the disease [36].

P53 is an independent predictor of disease-free survival, and the altered gene expression is a negative prognostic factor for overall survival of lung adenocarcinoma patients [37, 38]. P53 expression predicted sensitivity to cisplatin-based chemotherapy, whereas p53 mutation was found to be associated with resistance to cisplatin-based therapy. Increased P53 positivity conferred worse prognosis in untreated patients, and p53 mutation was also associated with worse prognosis. Steels et al. [39] showed that the mutated p53 gene leads to poorer survival in adenocarcinoma as well as in SCC in all stages of the disease. The increased sensitivity, by combining Rb2/p130, downstream of P53 suggests that it may be feasible to use a panel of genes such as p53 and Rb2/p130 as diagnostic markers of lung cancer.

### 2.4. BRCA1

BRCA1 is involved in transcription couples, nucleotide excision repair, and functions as a differential regulator of chemotherapy-induced apoptosis [40]. BRCA1 sensitizes cells to apoptosis induced by antimicrotubulin agents like taxanes and vinca alkaloids and also abrogates the effect of DNA-damaging agents like cisplatin and etoposide [41]. Low BRCA1 correlates with ERCC1 mRNA and predicts a favorable outcome in locally advanced NSCLC patients treated with cisplatin/gemcitabine, followed by surgery [42]. High BRCA1 predicts for resistance to cisplatin and possibly sensitivity to docetaxel [43]. BRCA1 overexpression conferred worse prognosis in untreated patients and suggested resistance to cisplatin-based chemotherapy [16].

### 2.5. RRM1

RRM1 is the regulatory component of ribonucleotide reductase, which catalyses the formation of deoxyribonucleotides from ribonucleotides, aiding with DNA synthesis and repair. Additionally, RRM1 mediates suppression of cell migration and tumour metastasis by inducing *PTEN*, a prominent tumour-suppressor gene responsible for attenuation of growth-factor pathway signalling. RRM1 is the predominant target of the nucleoside analogue gemcitabine. RRM1 has also been associated with differential survival outcomes in patients with NSCLC [44]. Increased RRM1 predicts for decreased tumor invasiveness and metastatic potential, therefore predicting for more indolent behavior, perhaps mediated through its direct correlation with phosphatase and tensin homolog (PTEN) protein expression [45]. High RRM1 levels therefore have a prognostic function. RRM1 expression correlates with expression of ERCC1, and patients whose tumors had high expression of RRM1 had longer survival compared with the low-expression group [46]. *RRM1* expression is a major predictor of disease response to gemcitabine/platinum chemotherapy [47].

### 2.6. ERCC1

ERCC1 is a rate-limiting protein in the NER and interstrand cross-link repair (ICL-R) pathways. It works by recognising and removing platinum adducts and by repairing interstrand DNA cross-links. NSCLC cell lines with increased *ERCC1 *have been shown to be resistant to platinum *in vitro* [48]. Patients with high expression of ERCC1 and surgically resected lung tumors have a better prognosis but no improvement of survival with chemotherapy, whereas patients with low expression of ERCC1 and surgically resected have a worse prognosis but a longer survival under adjuvant chemotherapy [49]. Determining ERCC1 expression in completely resected NSCLC could help select patients likely to benefit from additional platinum-based chemotherapy. ERCC1 expression in NSCLC is a controversial issue and needs extensive studies to exploit it as a biomarker.

### 2.7. Beta Tubulin

Microtubules are dynamic polymers that play a part in cell division. Aberrant expression of beta tubulin class III gene correlated with paclitaxel resistance in NSCLC cell lines. High beta tubulin expression correlated with shorter relapse-free and overall survival in untreated patients. No difference was shown in the responses of patients whose tumors had higher or lower beta tubulin expression [50]. Though studies have reported that high beta tubulin expression predicted resistance to vinorelbine [51], no improvement in survival was seen in patients assigned to either chemotherapy or observation in the low tubulin group, whereas in the high tubulin group, patients who received chemotherapy showed a trend towards overall improved survival, and hence, a significant treatment based on tubulin interaction could not be shown [50].

### 2.8. RB

The Rb tumor suppressor gene is located on chromosome 13q14 which takes part in the G1 check point of the cell cycle by inhibiting the transcription of certain genes whose protein products are necessary for DNA synthesis. The nonphosphorylated form of Rb forms a complex with E2F/DP1 key transcriptional factor and hinders the transition from G1 to S phase of the cell cycle and blocks cell duplication. Up to 30% of cytogenetic abnormalities of Rb gene have been detected by fluorescence *in situ* signaling in NSCLCs [52], and about 90% of SCLCs and 30% of NSCLCs lack active Rb protein [26]. RB in cooperation with other genetic abnormalities plays pivotal role in lung tumorigenesis, but do not represent a prognostic factor in NSCLCs. The 5-year survival rate in patients with normal versus reduced pRb expression was 55.1 versus 73%, the difference being nonsignificant [53]. Studies that delved into the prognostic value of this gene have had contradictory results. Xu et al. [54] and Caputi et al. [55] reported that when Rb is not expressed or expressed in lower degree, it correlated with poorer survival, whereas D'Amico et al. [56] in their elaborate study could not validate the prognostic value of RB.

### 2.9. AKT

AKT or protein kinase B is a serine threonine kinase that is activated by PI3K*α* and mediates PI3K signaling. Somatic mutations in AKT1 have been found in *≈*1% of all NSCLC in both adenocarcinoma and squamous cell carcinoma histology [57]. Preclinical data has shown that the presence of this mutation results in cellular transformations *in vitro* and *in vivo* [58]. As AKT activity regulates many processes in cancer, the AKT pathway has become an important therapeutic target. AKT activation promotes resistance to standard chemotherapy and radiotherapy, whereas inhibition of AKT signaling induces apoptosis and decreased growth of tumor cells dependent on elevated AKT signaling for cell survival and growth. In lung cancer, elevated AKT does not correlate with tumor stage or grade. Increased expression of phosphor AKT has been reported in preneoplastic lesions such as bronchial dysplasia, suggesting that AKT activation can be an early event in tumor progression and, thus, may represent a potentially important target for chemoprevention in individuals with high risk of lung cancer [59, 60]. 

A major recurrent mutation Glu17Lys has been observed in about 1% of NSCLC, and they have only been identified in squamous cell carcinoma. The Glu17Lys mutations occur in the AKT1 pleckstrin homology domain. This mutation alters the phosphoinositide binding pocket and leads to PI3K-independent protein kinase B activation [58]. *In vitro* studies suggest that the AKT1 E17K mutation is less sensitive than wild-type AKT1 to inhibitors by the experimental AKT inhibitor, VIII, a non-ATP competitive agent which requires a functional pleckstrin homology domain [58]. Allosteric inhibitors of protein kinase B are being tested in phase I trials.

The role of AKT1 mutations for selecting or prioritizing anticancer treatment is uncertain at this time. However, it should be noted that AKT1 mutations are usually found in wild-type tumors for EGFR, ALK, or other driver mutations.

### 2.10. PTEN

PTEN is a dual-specific lipid or protein phosphatase and acts as a tumor suppressor by negatively regulating PI3K/AKT pathway by dephosphorylating PIP3 [61]. Cancer-associated genomic alterations in PTEN result in PTEN inactivation, and hence, PI3K-AKT pathway is enhanced. Somatic mutations in PTEN have been found in 4–8% of NSCLC and are common in smokers with squamous cell histology. PTEN mutations occur in multiple exons, and no hotspots have been identified [62]. The common PTEN mutation is R233∗ which introduces premature stop codon into the PTEN gene. In preclinical studies, PTEN loss is associated with decreased sensitivity of EGFR mutant lung tumors to EGFR TKIs [63]. Clinical trials are assessing the efficacy of PI3K inhibitors in PTEN loss. Studies by Janku et al. as presented at the 2012 ASCO annual meeting showed that PTEN loss can be associated with increased PI3K/AKT/mTOR signaling and sensitivity to PI3K pathway inhibitors [64].

### 2.11. MET

The MET gene (MNNG-HOS) transforming gene, located on chromosome 7, encodes an RTK of the MET/RON family and plays an important role in PI3K-AKT-mTOR pathway as well as the RAS-RAF-MEK-ERK pathway. Aberrant signaling through MET receptor as with the case of malignancy promotes pleiotropic effects including growth, survival, invasion, migration, angiogenesis, and metastasis. MET mutations have been reported in NSCLC as well as SCLC [65, 66]. Overexpression of MET protein in tumor tissue relative to adjacent normal tissue occurs in 25–75% of NSCLC [67]. In a recent phase II study in which patients with NSCLC were randomized to MetMab (an anti-MET antibody) plus erlotinib versus erlotinib alone, increased expression of MET protein was associated with improved progression-free survival (HR = 0.53, *P* = 0.04: 1.5 months to 2.9 months) and overall survival (HR = 0.37, *P* = 0.002: 3.8 months to 12.6 months) in patients who received MetMAb and erlotinib [68]. MET amplifications play an important role in acquired resistance to EGFR inhibitors in patients with EGFR mutant tumors [69].

### 2.12. MEK1

MEK1, also known as MAP2K1, is a serine threonine protein kinase which plays a pivotal role in the MAP kinase signaling pathway and hence plays an important role in many cellular processes. Somatic mutations in MEK1 have been found in approximately 1% of all NSCLCs and are more common in adenocarcinoma histology [70]. The presence of MEK1 mutation is associated with *in vitro* resistance to EGFR TKIs but is found in wild-type tumors for EGFR, ALK, or any other driver mutations [71]. The common MEK1 mutations seen are Q56p at exon 2, K57N at exon 2 and D67N at exon 2.

### 2.13. HER2

HER2 belongs to RTK family that includes EGFR/ERBB1, HER2/ERBB2/NEU, HER3/ERBB3, and HER4/ERBB4. Though no ligand has been identified for HER2, it appears to be the preferential dimerization partner for all members of the ERBB family. Binding of ligand leads to heterodimerisation and activation of HER2 TK activity. Activated HER2 then phosphorylates its substrates leading to the activation of multiple downstream pathways including PI3K-AKT-mTOR and RAS-RAF-MEK-ERK pathways. HER2 mutations are detected in approximately 2–4% of NSCLC. The most common mutation is an in-frame insertion within exon 20. Though commonly found in never smokers with adenocarcinoma histology, HER2 mutations are also reported in other subsets of NSCLC [71]. HER2 mutations are nonoverlapping with oncogenic mutations found in NSCLC. HER2 amplifications do not usually coincide with HER2 mutations and have no important role as a predictive or prognostic marker contrarily to what is observed in breast. Preclinical data suggests that the presence of HER2 mutation is associated with primary resistance to the first generation EGFR TKIs, erlotinib, and gefitinib. However, cells expressing the HER2 exon 20 mutation are sensitive to the irreversible dual EGFR and HER2 TKIs, lapatinib, neratinib, and afatinib [72, 73].

### 2.14. CDKN2A

CDKN2A is a TS gene that regulates cell cycle progression through a G1/S restriction point by inhibiting CDK4 and CDK6/cyclin D-mediated phosphorylation of Rb. CDKN2A locus (9p21) encodes 2 proteins by alternative splicing: *α* transcript p16^INK4A^ which inhibits Rb phosphorylation and *β* transcript p14^ARF^ which stabilizes MDM2 and increases the availability of p53. Deregulated expression of p16^INK4A^ occurs in 43% of NSCLCs but is not related to the clinical stage of the tumors, but smoking was associated with aberrant expression of p16INK4A/pRB, suggesting that abnormalities in this network occur early in the development of NSCLC subset [74].

### 2.15. BRAF

BRAF is a serine threonine kinase that links RAS GTPase to downstream proteins of the MAPk family which control cell proliferation. In NSCLC, BRAF mutations are found in 1–3% of tumors, most of which are adenocarcinoma. Unlike melanoma, NSCLCs mostly harbor non-Val600Glu mutations including the Leu596Val mutation in the kinase domain [75–77]. BRAF mutations are associated with increased kinase activity and lead to constitutive activation of MAPK2 and MAPK3 [78]. The various BRAF mutations observed in NSCLC are as follows:

- G466V and G469A which occur within the highly conserved GXGXXG motif of the BRAF kinase domain.
- L597V which occurs within the kinase domain.
- V600E which occurs within the activation segment of the kinase domain.

BRAF mutations are mutually exclusive to EGFR and KRAS mutations and have been associated with decreased sensitivity to the EGFR TKI, gefitinib [78]. BRAF mutation is a powerful predictive marker and is evolving as a prognostic marker which can identify a subset of tumors that are sensitive to targeted therapies. Multiple BRAF inhibitors like PLX4032 (molecular inhibitor selective for BRAF), sorafenib (a multikinase inhibitor of RAF-1, BRAF, VEGF, and PDGF), and CI-1040 (a first-generation-specific inhibitor of MAP kinase) are currently under development. Clinical responses of patients harboring a BRAF V600E lung tumor-associated mutation to BRAF inhibitors are unknown at present. However, a phase I trial of the BRAF inhibitor, vemurafenib (PLX4032), showed >80% response rate in BRAF V600E positive melanoma [79]. In the follow-up randomized phase III trial comparing vemurafenib to dacarbazine in previously untreated, metastatic melanoma with the BRAF V600E mutation, vemurafenib improved rates of overall survival and progression-free survival [80]. Clinical responses of patients harboring a BRAF L597V, G469A, and G466V lung tumor associated mutation to BRAF inhibitors are unknown at present.

### 2.16. ALK

ALK encodes a tyrosine kinase receptor that is normally expressed in selected neuronal cell types. Several balanced translocations involving ALK have been discovered; however, all resulting chimeric proteins retain the ALK kinase domain. Majority of ALK fusion variants are comprised of EML4. Fusions of ALK with EML4 were found in NSCLC in 2007. ALK/EML4 fusion results from an inversion in short arm of chromosome 2 that juxtaposes the EML4 gene with the ALK gene. ALK/EML4 fusion results in protein oligomerisation and constitutive activation of the kinase [80]. Approximately 3–7% of lung tumors harbor ALK fusions. ALK fusions are more commonly found in light smokers (<10 pack years) and/or never smokers. ALK fusions are also associated with younger age and adenocarcinomas. 

Clinically the presence of ALK rearrangement is detected by FISH with an ALK break-apart probe. In the vast majority of NSCLC cases, ALK rearrangements are nonoverlapping with other oncogenic mutations [81]. In the initial phase I trial, patients whose tumors harbored an ALK fusion displayed a 57% radiographic response rate to the dual ALK/MET TKI, crizotinib [82]. The probability of progression-free survival (PFS) at 6 months was estimated to be around 72%. Based on these initial results, an international phase III trial randomizing patients with advanced lung cancer harboring ALK fusions to crizotinib versus standard chemotherapy after disease progression on first-line treatment is now ongoing. More recently, a retrospective study demonstrated that patients with ALK fusion-positive lung cancers have improved response rate and progression-free survival when treated with either pemetrexed monotherapy or combination therapy [83]. A superior median PFS (9 months) was observed for patients with ALK-positive lung cancer treated with pemetrexed compared to patients with EGFR mutant (PFS 5.5 months), KRAS mutant (PFS 7 months), or “triple negative” (ALK fusion negative, wild-type EGFR, and wild-type KRAS; PFS 4 months) lung cancer [84]. In addition, in a phase II nonrandomized study of the heat shock protein 90 (HSP-90) inhibitor, IPI-504, in patients with advanced lung cancer who previously progressed on EGFR TKI therapy, tumors from 3 patients were retrospectively found to have ALK rearrangements. 2 of these patients had partial response, while a third had prolonged stable disease (7.2 months, 24% decrease in tumor size [85].

In addition to mere ALK-EML4 rearrangements, which are sensitive to crizotinib, several second site mutations in ALK are also observed. The EML4-ALK L1152R mutation results in an amino acid substitution at position 1152 in ALK, from an L to R. L1152R has been detected in the tumor of a patient with ALK fusion-positive lung cancer who developed acquired resistance to the dual ALK/MET TKI crizotinib [86]. In addition to L1152R, additional nonoverlapping ALK kinase domain mutations L1196M and C1156Y which confer resistance to crizotinib have been detected in patients with ALK fusion-positive NSCLC [87]. ALK rearrangement is gaining more importance and is included in the gene testing panel for lung cancer.

### 2.17. PIK3CA

PI3K belongs to the family of lipid kinases involved in cell growth, proliferation, differentiation, motility and survival. It is a heterodimer composed of 2 subunits—an 85 Kda regulatory subunit p85 and a 110 Kda catalytic subunit PIK3CA code for one of the catalytic subunit p110*α*. PI3K converts PIP2 to PIP3 on the inner leaflet of the cell membrane. PIP3 recruits signaling proteins like AKT resulting in their increased activity. Somatic mutations in PI3KCA have been found in around 1–3% of NSCLC. These mutations usually occur within 2 hotspots within the helical domain of exon 9 and kinase domain of exon 20. When compared between adenocarcinoma and squamous cell carcinoma, PI3KA mutation is more common among adenocarcinoma and occurs in both smokers and nonsmokers. PIK3CA mutation can co-occur with EGFR mutations [88]. In addition, PIK3CA mutations have been detected in a small percentage (~5%) of EGFR mutant lung cancers with acquired resistance to EGFR TKI therapy [89]. The common PI3KCA mutations in lung cancer involve E542K, E545K, E545Q, H1047L, and H1047R.

### 2.18. ROS

ROS kinase is an RTK of the insulin receptor family. The normal function of ROS in humans is yet unclear; however, the production of variant mutant forms of ROS is widely reported in NSCLC. The expression of ROS gene is primarily restricted to distinct epithelial cells during embryonic development [84, 90–92]. ROS rearrangements have been recently shown to be involved in chromosomal translocations in lung cancer [93]. Elevated ROS expression is observed in both early- and late-stage lung tumors [94] suggesting its role in initiation or development rather than progression. ROS1 rearrangement defines a molecular subset of NSCLC whose clinical characteristics are similar as in patients with ALK rearrangements, and crizotinib shows clinical activity in NSCLC with ROS1 rearrangement [95].

### 2.19. FGFR1 

Fibroblast growth factor receptor 1 encodes a member of the FGFR TK family, which originally consists of 4 TKS, namely, FGFR1, 2, 3, and 4. FGFR TK plays important role in development and has been found to be deregulated in cancers either by amplifications, point mutations or translocations [96]. FGFR1 amplifications are predominantly seen among former or current smokers with squamous cell carcinoma (*≈*20%). Tumor cells with FGFR1 amplification are dependent on the activity of receptor, and hence, this dependency can be exploited therapeutically with FGFR inhibitors that have shown promising activity in preclinical models of lung cancer [97, 98]. 

### 2.20. DDR

Discoidin death receptor 2 is a member of the DDR family of receptor Tyr kinases that are stimulated by collagen rather than by peptide growth actors. Similar to integrin receptors, DDR2 plays a role in modulating cellular interactions with the extracellular matrix. DDR2 mutations have been found in 3-4 % of SCC of the lung but in <1% of lung tumors with squamous cell histology [99]. No hotspots have been identified, with mutations spanning both the kinase and discoidin domains [100]. Neither overexpression of DDR2 nor copy number alterations of the DDR2 locus (1q23) have been reported. The most common DDR2 mutation in SCC of lung is the S768R which occurs in the kinase domain. Mutant DDR2 proteins are transforming *in vitro*, and the cell lines harboring DDR2 mutations appear to be dependent on the mutant kinase activity for growth and survival. While there are limited data on the clinical characteristics of patients harboring DDR2 mutations, no significant association with sex, age, or smoking status has been found. 

Dasatinib had been shown to be an inhibitor of DDR2 in an *in vitro* screen [101], but dasatinib is a multiple kinase inhibitor. Squamous cell lung cancer cells expressing the I638F and L239R mutant receptors are sensitive to treatment with dasatinib. In addition, mouse xenografts derived from these cell lines regressed following treatment with dasatinib [99]. No clinical trials specifically targeting DDR2 mutations have been conducted. However, a DDR2 S768R mutation was found in the single squamous cell carcinoma of the lung that responded to treatment in an early-phase clinical trial of dasatinib and erlotinib. An overlapping EGFR mutation was not present [99]. While relatively uncommon, DDR2 mutations identify a subgroup of patients in whom treatment with an existing, well-characterized drug may prove beneficial.

Apart from the above-mentioned molecular alterations, polymorphisms in metabolic genes and estrogen are also having potential as biomarkers in lung carcinogenesis. In a previous study, we have observed reduced expression of estrogen in lung cancer patients which was authenticated by the increased expression of IL-6 and high CYP1A1 polymorphism. Estrogen was believed to be the fueling agent for cancer especially breast cancer. But its role in lung cancer was not explained in detail. The observation of reduction in estrogen in lung cancer patient's serum is a novel finding and was supported by the high rate of CYP1A1 genetic polymorphism and increased expression of IL-6 in serum [102]. It was reported that estradiol inhibits the production of proliferating cytokines including IL-6 and macrophage inhibitory factors. Estrogens are eliminated from the body by metabolically conversion to estrogenically inactive metabolites that are excreted in the urine and feces. Therefore, CYP1A1 activation might be the reason for the reduced estradiol and thereby increased expression of IL-6 in lung cancer. After detailed studies in a large cohort, serum levels of IL-6, estradiol, and genetic polymorphisms in CYP1A1 gene may also be included as biomarkers for early detection of lung cancer.

## 3. Conclusion

The present era belongs to personalized chemotherapy, and treatment choices based on molecular profiling are being studied intensely worldwide. This paper discusses various molecular alterations seen in lung cancer, and their predictive and prognostic implications are also being looked into. The usefulness of any molecular entity in selecting or prioritizing treatment in lung cancer is still controversial. However, with the advanced techniques like microarray, RT PCR and so forth, the most promising biomarkers of prediction and response can be prospectively validated.

## References

[1] Tran Y., Benbatoul K., Gorse K. (1998). Novel regions of allelic deletion on chromosome 18p in tumors of the lung, brain and breast. *Oncogene*.

[2] Sherbet G. W., Lakshi M. S. (1997). *The Genetics of Cancer*.

[3] Miura I., Graziano S. L., Jin Quan Cheng, Doyle L. A., Testa J. R. (1992). Chromosome alterations in human small cell lung cancer: frequent involvement of 5q. *Cancer Research*.

[4] Testa J. R., Siegfried J. M. (1992). Chromosome abnormalities in human non-small cell lung cancer. *Cancer Research*.

[5] Jarrard D. F., Bussemakers M. J. G., Bova G. S., Isaacs W. B. (1995). Regional loss of imprinting of the insulin-like growth factor II gene occurs in human prostate tissues. *Clinical Cancer Research*.

[6] Nakanishi H., Suda T., Katoh M. (2004). Loss of imprinting of PEG1/MEST in lung cancer cell lines. *Oncology Reports*.

[7] Dang T. P., Gazdar A. F., Virmani A. K. (2000). Chromosome 19 translocation, overexpression of Notch3, and human lung cancer. *Journal of the National Cancer Institute*.

[8] Sekido Y., Fong K. M., Minna J. D. (2003). Molecular genetics of lung cancer. *Annual Review of Medicine*.

[9] Klein G., Klein E. (2005). Surveillance against tumors—is it mainly immunological?. *Immunology Letters*.

[10] Heist R. S., Engelman J. A. (2012). SnapShot: non-small cell lung cancer. *Cancer Cell*.

[11] Pao W., Girard N. (2011). New driver mutations in non-small-cell lung cancer. *The Lancet Oncology*.

[12] Pao W., Chmielecki J. (2010). Rational, biologically based treatment of EGFR-mutant non-small-cell lung cancer. *Nature Reviews Cancer*.

[13] Soh J., Okumura N., Lockwood W. W. (2009). Oncogene mutations, copy number gains and mutant allele specific imbalance (MASI) frequently occur together in tumor cells. *PLoS ONE*.

[14] Li A. R., Chitale D., Riely G. J. (2008). Clinical testing experience and relationship to EGFR gene copy number and immunohistochemical expression. *Journal of Molecular Diagnostics*.

[15] Giovannetti E., Zucali P. A., Peters G. J. (2010). Association of polymorphisms in AKT1 and EGFR with clinical outcome and toxicity in non-small cell lung cancer patients treated with gefitinib. *Molecular Cancer Therapeutics*.

[16] Coate L. E., John T., Tsao M. S., Shepherd F. A. (2009). Molecular predictive and prognostic markers in non-small-cell lung cancer. *The Lancet Oncology*.

[17] Forbes S., Clements J., Dawson E. (2006). COSMIC 2005. *British Journal of Cancer*.

[18] Brose M. S., Volpe P., Feldman M. (2002). BRAF and RAS mutations in human lung cancer and melanoma. *Cancer Research*.

[19] Kosaka T., Yatabe Y., Onozato R., Kuwano H., Mitsudomi T. (2009). Prognostic implication of EGFR, KRAS, and TP53 gene mutations in a large cohort of Japanese patients with surgically treated lung adenocarcinoma. *Journal of Thoracic Oncology*.

[20] Riely G. J., Marks J., Pao W. (2009). KRAS mutations in non-small cell lung cancer. *Proceedings of the American Thoracic Society*.

[21] Mills N. E., Fishman C. L., Scholes J., Anderson S. E., Rom W. N., Jacobson D. R. (1995). Detection of K-ras oncogene mutations in bronchoalveolar lavage fluid for lung cancer diagnosis. *Journal of the National Cancer Institute*.

[22] Sugio K., Ishida T., Yokoyama H., Inoue T., Sugimachi K., Sasazuki T. (1992). ras Gene mutations as a prognostic marker in adenocarcinoma of the human lung without lymph node metastasis. *Cancer Research*.

[23] Camps C., Sirera R., Bremnes R. (2005). Is there a prognostic role of K-ras point mutations in the serum of patients with advanced non-small cell lung cancer?. *Lung Cancer*.

[24] Rosell R., Monzó M., Pifarré A. (1996). Molecular staging of non-small cell lung cancer according to K-ras genotypes. *Clinical Cancer Research*.

[25] Raponi M., Winkler H., Dracopoli N. C. (2008). KRAS mutations predict response to EGFR inhibitors. *Current Opinion in Pharmacology*.

[26] Rom W. N., Hay J. G., Lee T. C., Jiang Y., Tchou-Wong K. M. (2000). Molecular and genetic aspects of lung cancer. *American Journal of Respiratory and Critical Care Medicine*.

[27] Sigal A., Rotter V. (2000). Oncogenic mutations of the p53 tumor suppressor: the demons of the guardian of the genome. *Cancer Research*.

[28] Cho Y., Gorina S., Jeffrey P. D., Pavletich N. P. (1994). Crystal structure of a p53 tumor suppressor-DNA complex: understanding tumorigenic mutations. *Science*.

[29] Caron de Fromentel C., Soussi T. (1992). TP53 tumor suppressor gene: a model for investigating human mutagenesis. *Genes Chromosomes and Cancer*.

[30] Jassem E., Nikliński J., Rosell R. (2001). Types and localisation of p53 gene mutations: a report on 332 non-small cell lung cancer patients. *Lung Cancer*.

[31] May P., May E. (1999). Twenty years of p53 research: structural and functional aspects of the p53 protein. *Oncogene*.

[32] Gao W. M., Mady H. H., Yu G. Y. (2003). Comparison of p53 mutations between adenocarcinoma and squamous cell carcinoma of the lung: unique spectra involving G to A transitions and G to T transversions in both histologic types. *Lung Cancer*.

[33] Kishimoto Y., Murakami Y., Shiraishi M., Hayashi K., Sekiya T. (1992). Aberrations of the p53 tumor suppressor gene in human non-small cell carcinomas of the lung. *Cancer Research*.

[34] Cherneva R. V., Georgiev O. B., Petrov D. B., Dimova I. I., Toncheva D. I. (2009). Expression levels of p53 messenger RNA detected by real time PCR in tumor tissue, lymph nodes and peripheral blood of patients with non-small cell lung cancer—new perspectives for clinicopathological application. *Biotechnology and Biotechnological Equipment*.

[35] Lubin R., Zalcman G., Bouchet L. (1995). Serum p53 antibodies as early markers of lung cancer. *Nature Medicine*.

[36] Piyathilake C. J., Frost A. R., Manne U., Weiss H., Heimburger D. C., Grizzle W. E. (2003). Nuclear accumulation of p53 is a potential marker for the development of squamous cell lung cancer in smokers. *Chest*.

[37] Quinlan D. C., Davidson A. G., Summers C. L., Warden H. E., Doshi H. M. (1992). Accumulation of p53 protein correlates with a poor prognosis in human lung cancer. *Cancer Research*.

[38] Tsao M. S., Aviel-Ronen S., Ding K. (2007). Prognostic and predictive importance of p53 and RAS for adjuvant chemotherapy in non-small-cell lung cancer. *Journal of Clinical Oncology*.

[39] Steels E., Paesmans M., Berghmans T. (2001). Role of p53 as a prognostic factor for survival in lung cancer: a systematic review of the literature with a meta-analysis. *European Respiratory Journal*.

[40] Wachters F. M., Wong L. S. M., Timens W., Kampinga H. H., Groen H. J. M. (2005). ERCC1, hRad51, and BRCA1 protein expression in relation to tumour response and survival of stage III/IV NSCLC patients treated with chemotherapy. *Lung Cancer*.

[41] Rosell R., Skrzypski M., Jassem E. (2007). BRCA1: a novel prognostic factor in resected non-small-cell lung cancer. *PLoS ONE*.

[42] Wang L., Wei J., Qian X. (2008). ERCC1 and BRCA1 mRNA expression levels in metastatic malignant effusions is associated with chemosensitivity to cisplatin and/or docetaxel. *BMC Cancer*.

[43] Aggarwal C., Somaiah N., Simon G. R. (2010). Biomarkers with predictive and prognostic function in non-small cell lung cancer: ready for prime time?. *Journal of the National Comprehensive Cancer Network*.

[44] Rosell R., Danenberg K. D., Alberola V. (2004). Ribonucleotide reductase messenger RNA expression and survival in gemcitabine/cisplatin-treated advanced non-small cell lung cancer patients. *Clinical Cancer Research*.

[45] Gautam A., Li Z. R., Bepler G. (2003). RRM1-induced metastasis suppression through PTEN-regulated pathways. *Oncogene*.

[46] Zheng Z., Chen T., Li X., Haura E., Sharma A., Bepler G. (2007). DNA synthesis and repair genes RRM1 and ERCC1 in lung cancer. *New England Journal of Medicine*.

[47] Bepler G., Kusmartseva I., Sharma S. (2006). RRM1 modulated in vitro and in vivo efficacy of gemcitabine and platinum in non-small-cell lung cancer. *Journal of Clinical Oncology*.

[48] Altaha R., Liang X., Yu J. J., Reed E. (2004). Excision repair cross complementing-group 1: gene expression and platinum resistance. *International Journal of Molecular Medicine*.

[49] Olaussen K. A., Dunant A., Fouret P. (2006). DNA repair by ERCC1 in non-small-cell lung cancer and cisplatin-based adjuvant chemotherapy. *New England Journal of Medicine*.

[50] Sève P., Mackey J., Isaac S. (2005). Class III *β*-tubulin expression in tumor cells predicts response and outcome in patients with non-small cell lung cancer receiving paclitaxel. *Molecular Cancer Therapeutics*.

[51] Sève P., Lai R., Ding K. (2007). Class III *β*-tubulin expression and benefit from adjuvant cisplatin/vinorelbine chemotherapy in operable non-small cell lung cancer: analysis of NCIC JBR.10. *Clinical Cancer Research*.

[52] Wistuba I. I., Gazdar A. F., Minna J. D. (2001). Molecular genetics of small cell lung carcinoma. *Seminars in Oncology*.

[53] Sugio K., Tsukamoto S., Ushijima C. (2001). Clinical significance of the Rb expression in adenocarcinoma of the lung. *Anticancer Research*.

[54] Xu H. J., Quinlan D. C., Davidson A. G. (1994). Altered retinoblastoma protein expression and prognosis in early-stage non-small-cell lung carcinoma. *Journal of the National Cancer Institute*.

[55] Caputi M., Groeger A. M., Esposito V. (2002). Loss of pRb2/p130 expression is associated with unfavorable clinical outcome in lung cancer. *Clinical Cancer Research*.

[56] D'Amico T. A., Massey M., Herndon J. E., Moore M. B., Harpole D. H., Benfield J. R. (1999). A biologic risk model for stage I lung cancer: immunohistochemical analysis of 408 patients with the use of ten molecular markers. *Journal of Thoracic and Cardiovascular Surgery*.

[57] Bleeker F. E., Felicioni L., Buttitta F. (2008). AKT1E17K in human solid tumours. *Oncogene*.

[58] Carpten J. D., Faber A. L., Horn C. (2007). A transforming mutation in the pleckstrin homology domain of AKT1 in cancer. *Nature*.

[59] Tsao A. S., McDonnell T., Lam S. (2003). Increased phospho-AKT (Ser473) expression in bronchial dysplasia: implications for lung cancer prevention studies. *Cancer Epidemiology Biomarkers and Prevention*.

[60] Balsara B. R., Pei J., Mitsuuchi Y. (2004). Frequent activation of AKT in non-small cell lung carcinomas and preneoplastic bronchial lesions. *Carcinogenesis*.

[61] Davidson L., MacCario H., Perera N. M. (2010). Suppression of cellular proliferation and invasion by the concerted lipid and protein phosphatase activities of PTEN. *Oncogene*.

[62] Jin G., Kim M. J., Jeon H. S. (2010). PTEN mutations and relationship to EGFR, ERBB2, KRAS, and TP53 mutations in non-small cell lung cancers. *Lung Cancer*.

[63] Sos M. L., Koker M., Weir B. A. (2009). PTEN loss contributes to erlotinib resistance in EGFR-mutant lung cancer by activation of akt and EGFR. *Cancer Research*.

[64] Janku F., Jennifer J. W., Shannon N. W. (2011). PI3K/AKT/mTOR inhibitors in patients with breast and gynecologic malignancies harboring *PIK3CA* mutations. *Journal of Clinical Oncology*.

[65] Ma P. C., Kijima T., Maulik G. (2003). c-MET mutational analysis in small cell lung cancer: novel juxtamembrane domain mutations regulating cytoskeletal functions. *Cancer Research*.

[66] Kong-Beltran M., Seshagiri S., Zha J. (2006). Somatic mutations lead to an oncogenic deletion of Met in lung cancer. *Cancer Research*.

[67] Benedettini E., Sholl L. M., Peyton M. (2010). Met activation in non-small cell lung cancer is associated with de novo resistance to EGFR inhibitors and the development of brain metastasis. *American Journal of Pathology*.

[68] Spigel D. R., Ervin T. J., Ramlau R. (2011). Final efficacy results from OAM4558g, a randomized phase II study evaluating MetMAb or placebo in combination with erlotinib in advanced NSCLC. *Journal of Clinical Oncology*.

[69] Bean J., Brennan C., Shih J. Y. (2007). MET amplification occurs with or without T790M mutations in EGFR mutant lung tumors with acquired resistance to gefitinib or erlotinib. *Proceedings of the National Academy of Sciences of the United States of America*.

[70] Marks J. L., Gong Y., Chitale D. (2008). Novel MEK1 mutation identified by mutational analysis of epidermal growth factor receptor signaling pathway genes in lung adenocarcinoma. *Cancer Research*.

[71] Buttitta F., Barassi F., Fresu G. (2006). Mutational analysis of the HER2 gene in lung tumors from Caucasian patients: mutations are mainly present in adenocarcinomas with bronchioloalveolar features. *International Journal of Cancer*.

[72] Wang S. E., Narasanna A., Perez-Torres M. (2006). HER2 kinase domain mutation results in constitutive phosphorylation and activation of HER2 and EGFR and resistance to EGFR tyrosine kinase inhibitors. *Cancer Cell*.

[73] Li D., Ambrogio L., Shimamura T. (2008). BIBW2992, an irreversible EGFR/HER2 inhibitor highly effective in preclinical lung cancer models. *Oncogene*.

[74] Gorgoulis V. G., Zacharatos P., Kotsinas A. (1998). Alterations of the p16-pRb pathway and the chromosome locus 9p21-22 in non-small-cell lung carcinomas: relationship with p53 and MDM2 protein expression. *American Journal of Pathology*.

[75] Davies H., Bignell G. R., Cox C. (2002). Mutations of the BRAF gene in human cancer. *Nature*.

[76] Sasaki H., Kawano O., Endo K. (2006). Uncommon V599E BRAF mutations in Japanese patients with lung cancer supported by the AstraZeneca Research grant, 2004. *Journal of Surgical Research*.

[77] Naoki K., Chen T. H., Richards W. G., Sugarbaker D. J., Meyerson M. (2002). Missense mutations of the BRAF gene in human lung adenocarcinoma. *Cancer Research*.

[78] Gandhi J., Zhang J., Xie Y. (2009). Alterations in genes of the EGFR signaling pathway and their relationship to EGFR tyrosine kinase inhibitor sensitivity in lung cancer cell lines. *PLoS ONE*.

[79] Flaherty K. T., Puzanov I., Kim K. B. (2010). Inhibition of mutated, activated BRAF in metastatic melanoma. *New England Journal of Medicine*.

[80] Chapman P. B., Hauschild A., Robert C. (2011). Improved survival with vemurafenib in melanoma with BRAF V600E mutation. *New England Journal of Medicine*.

[81] Soda M., Choi Y. L., Enomoto M. (2007). Identification of the transforming EML4-ALK fusion gene in non-small-cell lung cancer. *Nature*.

[82] Kwak E. L., Bang Y. J., Camidge D. R. (2010). Anaplastic lymphoma kinase inhibition in non-small-cell lung cancer. *New England Journal of Medicine*.

[83] Camidge D. R., Kono S. A., Lu X. (2011). Anaplastic lymphoma kinase gene rearrangements in non-small cell lung cancer are associated with prolonged progression-free survival on pemetrexed. *Journal of Thoracic Oncology*.

[84] Sonnenberg E., Godecke A., Walter B., Bladt F., Birchmeier C. (1991). Transient and locally restricted expression of the ros1 protooncogene during mouse development. *EMBO Journal*.

[85] Sequist L. V., Gettinger S., Senzer N. N. (2010). Activity of IPI-504, a novel heat-shock protein 90 inhibitor, in patients with molecularly defined non-small-cell lung cancer. *Journal of Clinical Oncology*.

[86] Sasaki T., Koivunen J., Ogino A. (2011). A novel ALK secondary mutation and EGFR signaling cause resistance to ALK kinase inhibitors. *Cancer Research*.

[87] Choi Y. L., Soda M., Yamashita Y. (2010). EML4-ALK mutations in lung cancer that confer resistance to ALK inhibitors. *New England Journal of Medicine*.

[88] Kawano O., Sasaki H., Endo K. (2006). *PIK3CA* mutation status in Japanese lung cancer patients. *Lung Cancer*.

[89] Sequist L. V., Waltman B. A., Dias-Santagata D. (2011). Genotypic and histological evolution of lung cancers acquiring resistance to EGFR inhibitors. *Science Translational Medicine*.

[90] Sonnenberg-Riethmacher E., Walter B., Riethmacher D., Gödecke S., Birchmeier C. (1996). The c-ros tyrosine kinase receptor controls regionalization and differentiation of epithelial cells in the epididymis. *Genes and Development*.

[91] Liu Z. Z., Wada J., Kumar A., Carone F. A., Takahashi M., Kanwar Y. S. (1996). Comparative role of phosphotyrosine kinase domains of c-ros and c-ret protooncogenes in metanephric development with respect to growth factors and matrix morphogens. *Developmental Biology*.

[92] Chen J., Zong C. S., Wang L. H. (1994). Tissue and epithelial cell-specific expression of chicken proto-oncogene c-ros in several organs suggests that it may play roles in their development and mature functions. *Oncogene*.

[93] Rikova K., Guo A., Zeng Q. (2007). Global survey of phosphotyrosine signaling identifies oncogenic kinases in lung cancer. *Cell*.

[94] Bonner A. E., Lemon W. J., Devereux T. R., Lubet R. A., You M. (2004). Molecular profiling of mouse lung tumors: association with tumor progression, lung development, and human lung adenocarcinomas. *Oncogene*.

[95] Bergethon K., Shaw A. T., Ou S.-H. I. (2012). ROS1 rearrangements define a unique molecular class of lung cancers.

[96] Turner N., Grose R. (2010). Fibroblast growth factor signalling: from development to cancer. *Nature Reviews Cancer*.

[97] Weiss J., Sos M. L., Seidel D. (2010). Frequent and focal FGFR1 amplification associates with therapeutically tractable FGFR1 dependency in squamous cell lung cancer. *Science Translational Medicine*.

[98] Dutt A., Ramos A. H., Hammerman P. S. (2011). Inhibitor-sensitive fgfr1 amplification in human non-small cell lung cancer. *PLoS ONE*.

[99] Hammerman P. S., Sos M. L., Ramos A. H. (2011). Mutations in the DDR2 kinase gene identify a novel therapeutic target in squamous cell lung cancer. *Cancer Discovery*.

[100] Ichikawa O., Osawa M., Nishida N., Goshima N., Nomura N., Shimada I. (2007). Structural basis of the collagen-binding mode of discoidin domain receptor 2. *EMBO Journal*.

[101] Day E., Waters B., Spiegel K. (2008). Inhibition of collagen-induced discoidin domain receptor 1 and 2 activation by imatinib, nilotinib and dasatinib. *European Journal of Pharmacology*.

[102] Sreelekha T. T., Rajesh M., Anil Kumar V., Madhavan J., Balaram P. (2010). CYP1A1m2 polymorphisms regulate estrogen and interleukin-6 in lung cancer. *Molecular Medicine Reports*.

